# Microplastics as Emerging Cotracers in Groundwater Quality Assessments

**DOI:** 10.1021/acsestwater.6c00063

**Published:** 2026-05-30

**Authors:** Barbara Zambelli, Emma Pemberton, Stefano Viaroli, Stefan Krause, Roberto Giannecchini, Viviana Re

**Affiliations:** † Earth Sciences Department, University of Pisa, Via Santa Maria 53, 56126 Pisa, Italy; ‡ Chief Scientist Group, Environment Agency, Red Kite House, Howbery Park, Bristol BS1 5AH, U.K.; § School of Geography, Earth and Environmental Sciences, 1724 University of Birmingham, Edgbaston, Birmingham B15 2TT, U.K.; ∥ Laboratory of the Ecology of Natural and Anthropised Hydrosystems (LEHNA), University of Lyon 1, 69622 Villeurbanne Cedex, France

## Abstract

This study evaluates the feasibility of using microplastics (MPs) as cotracers within multitracer hydrogeological frameworks, alongside established tools such as water-stable isotopes, major and trace elements, and chemicals of emerging concern. Given that plastic production expanded globally only after the 1950s, MPs can serve as event markers delimiting recent recharge episodes or, when detected in older groundwater, as indicators of mixing or artificial recharge. MP properties including particle size, polymer type, shape, and density carry information about particulate transport dynamics and anthropogenic influence that dissolved tracers cannot provide. The utility of MPs as cotracers is particularly relevant in young groundwater systems directly connected to surface environments. Key limitations include contamination risks, the influence of artificial recharge on signal interpretation, and the cost and complexity of MP characterization. Integrating MP with hydrochemical and isotopic data sets, developing context-specific sampling protocols, and prioritizing open data sharing would advance this emerging methodological approach.

## Graphical abstract

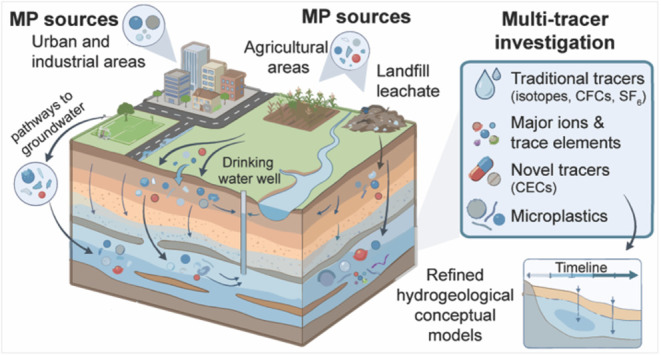

## Introduction

Plastics are versatile, cost-effective, and durable materials whose ubiquity in modern life^1^ has driven exponential production growth, approximately 10% annually since the 1950s,^2^ totaling over 9 billion tons globally in the past 50 years.^3^ This production surge, coupled with mismanaged plastic waste,^4^ plastics that reach end-of-life and are not recycled, incinerated, or kept permanently confined in sealed landfills,^5^ has resulted in the continuous environmental release of macro- and microplastics (MPs, particles <5 mm), through both primary production and secondary fragmentation.^6^ Despite two decades of research progress,^7^ MPs remain a critical gap in plastics lifecycle assessment,^8^ particularly regarding their environmental fluxes and fate.

The relevance of investigating MPs in groundwater is 2-fold. First, groundwater accounts for 99% of the liquid freshwater on the planet, providing a safe drinking water resource for half of the global population and serving 38% of the world’s irrigated areas.^9^ Groundwater also acts as an underground reservoir, supporting the baseflow of rivers and streams during dry periods, sustaining wetlands and other groundwater-dependent ecosystems.^10^ With demand projected to increase substantially by 2050^9^ alongside climate-driven pressures, protecting groundwater quality is paramount.^10^ Second, despite its self-depuration capacity, groundwater can be considered a major long-term contamination sink due to its typically slow movement and long residence times.^11,12^ Mitigating MPs contamination in groundwater is especially challenging given how dispersed their sources are in space and time and how pervasive and persistent the pollutant is.^7^ The capacity of particulate material to absorb and transport microorganisms inside aquifers is already highlighted in the literature, pointing to the relevance of research on suspended material in subsurface contamination hydrology.^13^

This paper pioneers a novel conceptual framework: integrating MPs as environmental cotracers within multitracer groundwater quality investigations. While previous MP groundwater studies have focused primarily on detection and quantification, this work positions MPs alongside established tracers such as stable isotopes and novel tracers like chemical contaminants to elucidate complex contaminant transport and groundwater mixing processes.

The use of environmental (passive) particulate groundwater tracers is still underexplored. Most studies on particulate tracers focus on artificial tracer tests, in which biological particles (e.g., microorganisms, yeast, and spores), natural colloids, suspended sediments, or fluorescent microspheres are injected into groundwater to investigate the aquifer’s vulnerability to contamination, particle transport and attenuation effects, and transfer parameters.^13–18^ MPs are unique to this role since existing particulate tracers are either injected, thus artificial and logistically demanding, or biologically active, being subject to growth and dye-off and regulatory concerns. MPs are the only particulate tracers that are (i) passively largely distributed in the environment,^19^ (ii) persistent over hydrogeological time scales, (iii) physically identifiable by polymer type and unambiguously anthropogenic,^20^ and (iv) capable of carrying source-specific chemical signatures.^21,22^

MPs’ diverse sizes, polymeric compositions, and production timelines may reveal valuable information about aquifer permeability, preferential flow pathways, recharge time scales, and pollution source identification, complementing traditional hydrogeological tracers in ways not previously explored in the literature. This paper, therefore, explores both the conceptual foundations for using MPs as groundwater passive cotracers and the methodological requirements necessary to operationalize this approach, advancing both fundamental understanding and practical application in groundwater quality assessment.

## Background

MPs were previously proposed by Pittroff et al. to be used as a potential environmental process tracer for driving riverbed dynamics, exploring how different processes, both natural and artificial, affect MP retention and sediment dynamics.^23^ Researchers have also proposed the use of MPs as tracers in different surface-aquatic systems. Van Sebille et al. suggested the use of MPs as tracers in marine environments to investigate mechanisms related to the functioning of currents, eddies, and water mixing.^24^ Rohais et al. proposed the use of MPs in riverine systems to characterize fluvial residence time and reservoir dynamics.^25^ Drummond et al. demonstrated that hyporheic exchange in riverine systems significantly influences MPs fate and transport, highlighting the use of MPs for understanding subsurface transport processes and particle trapping dynamics in saturated sediments.^26,27^ In addition to the role of hyporheic exchange in determining the fate and transport of MPs, Loui et al. emphasized the significance of bank filtration and its impacts on MP fate and transport in the subsurface.^28^

In groundwater, Goeppert and Goldscheider were the first ones to use the nomenclature “MPs” applied to carboxylate polystyrene microspheres (<5 μm) used as an artificial tracer, comparing their transport with uranine dye tracer in an alluvial aquifer in Germany.^29^ Before that, Schiperski et at. used a multitracer testing to identify processes that influence transport and attenuation of particles within a karst aquifer using 1-μm fluorescent particles (polystyrene and silicate, both plain and carboxylate) and fluorescent dye uranine.^16^ It is important to note that fluorescent microspheres have been used as artificial tracers in subsurface hydrology since the 1980s, mostly to investigate microbial and colloid transport.^15,30^ The microspheres were manufactured in a variety of sizes (usually smaller than 10 μm) and were commonly made of polystyrene coated with fluorescent dye.^31^

To date, the potential for using MPs as environmental tracers in groundwater has remained largely unexplored. We therefore explore the foundations of classic and novel groundwater tracers and their applications, including groundwater mixing assessment, recharge time scales and residence time, and contaminant transport. Finally, we discuss the opportunities and the limitations for including MPs in a multitracer groundwater quality assessment.

### Types of Groundwater Tracers

Groundwater tracers can be defined as substances, either dissolved or suspended, that can be identified and measured in groundwater. Their flow behavior in the aquifer can be used to infer aquifer properties as well as the fate and transport of the groundwater itself.^32^ An ideal tracer is mobile and has good dispersion characteristics, it is largely unreactive (conservative), is easily recognized and measured, and its transport is not strongly affected by interaction with the soil or aquifer matrix.^33,34^

When the target substance, of either natural (geogenic) or anthropogenic origin, is not intentionally added to groundwater, it is termed an environmental or passive tracer. Variations in their abundance can be utilized to trace mechanisms, pathways, and time scales of environmental processes,^33^ such as mixing between different water sources, processes, and rates of groundwater recharge, estimating groundwater age (time span between infiltration and sampling), measuring groundwater flow rates, and identifying sources of contaminants, among others.^35,36^ Environmental tracers allow for exploring large temporal and spatial scales of study and are therefore suitable and useful for determining the spatial variability of otherwise hard-to-observe underground systems. They are usually associated with local-to-regional studies.

Geogenic environmental tracers include various environmental isotopes. Stable isotopes of the water molecule, such as δ^18^O and δ^2^H, can be used to trace recharge processes and location due to isotope fractionation.^37^ Radioactive isotopes, such as δ^35^S (used to date groundwater that is about 150 to 200 days old), and δ^39^Ar (50 to 1000 years old), can serve as tracers for inferring groundwater flow time scales and geochemical reactions whenever decay is the main driver of concentration changes.^33,35^ Radiogenic or accumulating tracers such as δ^4^He and δ^222^Rn are produced by radioactive decay and accumulate in the subsurface, providing valuable information on groundwater residence time and helping in the identification of groundwater discharge zones into surface waters.^38,39^

Environmental tracers described as event markers are substances not produced or consumed in the subsurface that have a variable and well-known history of concentration changes in groundwater over time. They are useful to provide information on groundwater age. A classic example is δ^3^H; atmospheric concentrations increased in the 1950s and 1960s, resulting from nuclear bomb testing, and have decreased since then. Because of that, in the absence of other sources of contamination, the presence of δ^3^H in groundwater provides evidence of recharge after the 1950s.^40^ When the ratio of δ^3^H/^3^He is analyzed, it is possible to date groundwater between 0.1 and 50 years old.^41^ The same pattern can be observed for δ^36^Cl and δ^14^C, with both being used to indicate the contribution to groundwater recharge of precipitation after the 1950s–1960s.

Due to industrial activities since the mid-20th century, atmospheric concentrations of δ^85^Kr, chlorofluorocarbons (CFCs), and hexafluoride (SF_6_) increased, providing a useful signal to date young groundwater in areas where current contamination levels can be neglected - from 1950 to present for δ^85^Kr, CFC-11, CFC-12, and from 1970 to present for CFC-113 and SF_6_.^42,43^ Event markers also include environmental chemicals, like δ^3^H for landfill leaching, and other anthropogenic contaminants in case of creeping release or accidents.^33,35^

In this context, if found in groundwater, MPs could theoretically be considered an event marker, since the maximum groundwater age can be set to the time of polymer creation. Figure 1 presents the timeline of discovery and annual production of each polymer. The full data set and references are presented as Supporting Information. Alternatively, if MPs are found in groundwater known to be older than the polymer creation, this could indicate a faster contamination path and thus groundwater mixing. Identifying groundwater mixing could help us to understand groundwater preferential flows and possible contamination sources, a crucial step in mitigating exposure risks to that contaminant.

**Figure 1:**
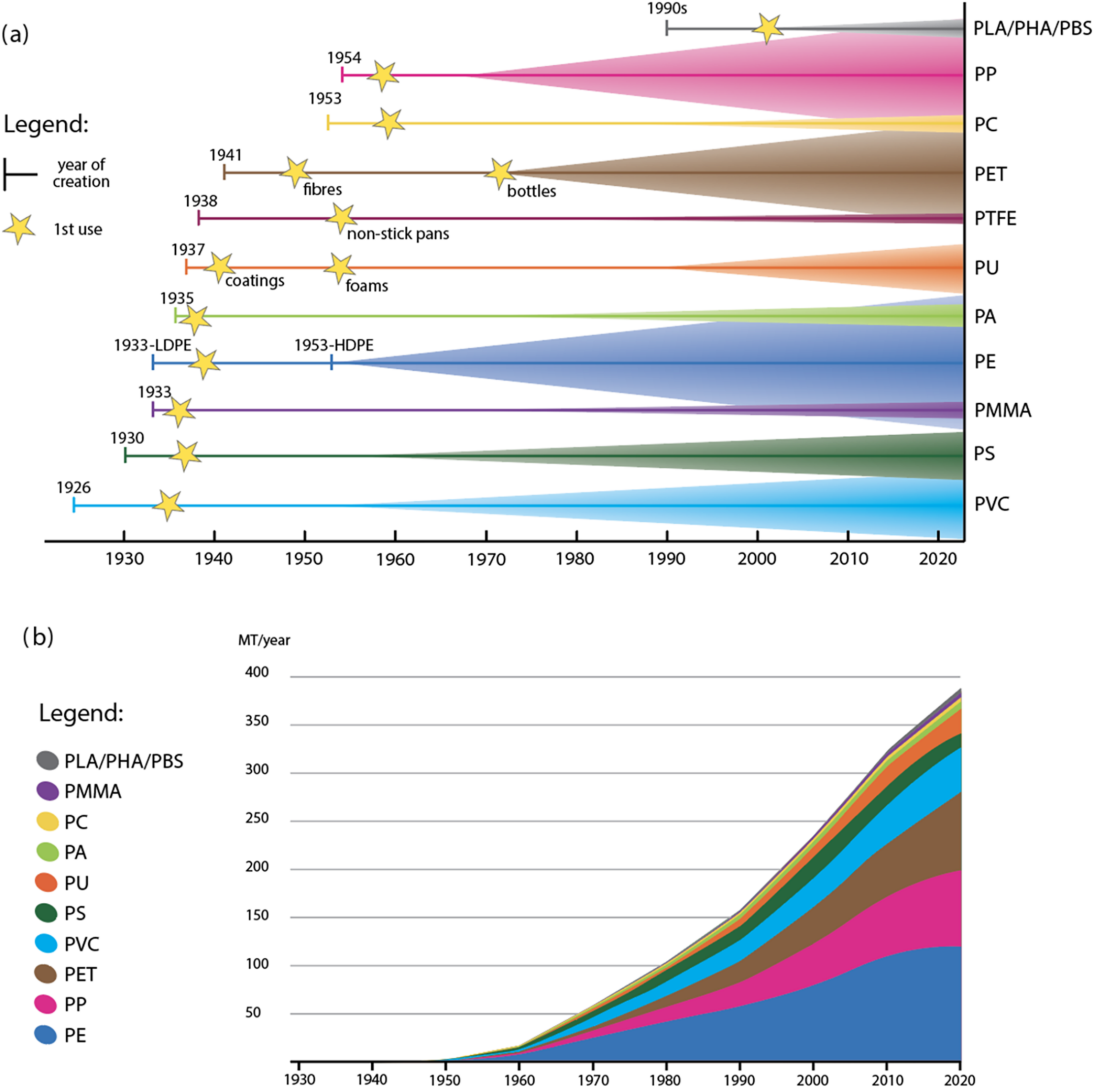
(a) Timeline of discovery and evolution of production of different polymers, with the year of creation and the first use marked. The width of the funnel indicates the global volume of production over decades. (b) Global production in Million Metric Tons per year of different polymers. (PE: polyethylene, PP: polypropylene, PET: polyethylene terephthalate, PVC: polyvinyl chloride, PS: polystyrene, PU: polyurethane, PA: polyamide, PC: polycarbonate, PMMA: poly­(methyl methacrylate), PLA, PHA, PBS: biodegradable plastics, PTFE: polytetrafluoroethylene).

### Multitracer Pollution Assessment and Novel Tracers

To effectively address pollution risks in groundwater, it is crucial to develop our understanding of pollution sources, their environmental fate, and transport pathways. Using multitracer approaches in groundwater quality assessment increases the robustness of generated conceptual groundwater models, improving the likelihood of identifying pollution sources and pathways, thus boosting the potential for effective pollution remediation and control. However, a notable disadvantage of a multitracer approach is the increment in overall costs and time for analysis.

Multitracer approaches for groundwater quality assessment have previously been applied to target nitrate^44,45^ and sulfate pollution,^46^ groundwater discharge and residence times,^47^ groundwater-lake interactions,^48^ and urban pollution sources.^49^ The use of chemicals of emerging concern (CECs) as cotracers has previously been proposed, along with traditional environmental tracers such as stable water isotopes, to gain an understanding of contamination sources and contaminant transport inside aquifers.^10,50–52^ These tracers have been described as “novel tracers”, referring to the recent development of their analytical quantification methods, measuring changes in concentration of these substances over time and space.

Crayol and colleagues (2024) argue that, due to differences in environmental persistence, some CECs could be used as proxies to evaluate groundwater residence time.^53^ For example, substances considered relatively persistent in aquatic environments, such as the herbicides simazine and fenuron, tend to be considered as legacy contaminants, whereas more biodegradable substances, such as caffeine, may indicate more recent infiltration and a shorter residence time. In this sense, MPs could also be used as novel groundwater cotracers, helping to identify pollution sources and providing information on physical aquifer properties.

### MPs in Groundwater: the Global Evidence

Understanding the occurrence of MPs in groundwater is paramount to their use as anthropogenic environmental tracers. Since the publication of the first paper addressing MP pollution in groundwater, there has been an increase in the number of papers exploring various aspects of MP occurrence in groundwater; however, only a fraction of them have provided field data on MPs in groundwater.^54–56^ Most of these studies were conducted in China, South Korea, and India, and a few others in Italy, Germany, Slovenia, Greece, the USA, Mexico, Iran, and Australia.^57^

In 2024, data for MPs in groundwater were reported in the scientific literature in less than 400 samples worldwide.^19^ The number of samples collected in individual studies varied between 3^58^ and 36^59^, and sample volumes ranged from 1 L^58–63^ to 1000 L^54^ of water. Where MPs were identified, their concentrations varied greatly, ranging from 0.0013 to 6832 particles per liter.^54,64^ It remains unclear to what degree the observed range in reported particle concentrations is affected by sampling- and/or analysis-related issues and environmental controls. Cha et al. explored how groundwater sample volume impacts MP detection, observing that MP concentration decreases with increasing sampling volume.^65^ Separation and extraction techniques, analytical methods employed, equipment detection limits, and thresholds used may cause methodological bias in the MP detection. In addition, aquifer vulnerability to pollution depends mostly on the hydrogeological setting, depth to groundwater, and thickness and permeability of the vadose zone; moreover, the input of MP pollution can be heavily influenced by land use around the sampling area.

The type of aquifer sampled affects groundwater residence time and contaminant fate and transport, thus the likelihood of finding MPs, their abundance, and size.^56^ So far, MPs have been identified in shallow granular aquifers described by the authors as sedimentary,^59^ alluvial,^66,67^ coastal,^60–62^ unconsolidated sediments,^54^ and weathered rocks^68^ in depths up to 34 m.^60^ In karst aquifers,^69–71^ groundwater was sampled from springs and conduits, whereas in fractured volcanic and crystalline aquifers, samples were collected in depths up to 240 m.^65,72–74^

### Proposal of a New Framework: MPs as Groundwater Cotracers

This paper proposes the use of MPs as cotracers in the ensemble of groundwater quality monitoring constituents, focusing on young/modern groundwater systems, given that these systems are the most vulnerable to anthropogenic contamination. Wherever natural recharge processes prevail, MPs contamination in groundwater is expected to mostly affect aquifers recharged since the 1950′s—the decade when plastics became widely available commercially.

Young groundwater is defined as groundwater less than 100 years old, accounting for the vast majority of groundwater found in depths up to 250 m below land surface, and is the main supplier of drinking water worldwide.^75^ Even though old/fossil groundwater represents a larger volume when compared to young groundwater, the latter represents the active part of the water cycle, discharging into streams and connecting to rivers, lakes, and oceans. This connection with the surface environment allows the surface-groundwater exchange and facilitates MP migration through sediments and soils and their ultimate integration in groundwater, resulting in a greater exposure to anthropogenic contamination when compared to old/fossil groundwater.^27,75–77^

Collecting supporting auxiliary field data, such as water-stable isotopes, for joint interpretation alongside MP information can help to trace recharge sources and processes, potentially helping to identify sources of MP pollution. Knowledge of different major and trace elements and CECs present may help to understand anthropogenic contamination sources and groundwater residence time.^10,33,50,53^ The combination of the aforementioned data with information on MPs found in groundwater such as polymer type, concentration, and size could be instrumental in understanding MPs sources and behavior in the aquifer.^56^ Such comprehensive analysis could be incorporated into the hydrogeological conceptual modeling, potentially elucidating MP sources and pathways through environmental compartments toward groundwater.

In fact, Perraki et al.^78^ used major ions to characterize seawater intrusion, the impact of agricultural activities, and MP pollution in the subsurface environment. Kim et al. found a positive correlation between major ions and MP concentration, suggesting that in their study area, environmental ions could be used as proxies for MP contamination.^74^ Alvarado-Zambrano et al., working in a shallow coastal aquifer, found a positive correlation between the abundance of MPs and total dissolved solids, electric conductivity, turbidity, and chlorine concentration in groundwater, identifying marine intrusion as a possible source of MPs in the aquifer.^60^ Panno et al. reported a positive correlation of CECs and MP concentrations.^71^ In different matrices (road dust and marine environment), plastic additives were used as proxies for MP contamination.^21,22^

If interpreted as tracers, MPs in aquifers could be used to answer a range of questions. MP transport in the subsurface is governed by a different and decoupled set of physical controls compared to natural suspended sediments. For natural colloids, size and density broadly covary and size exclusion governs breakthrough order, with coarser particles being recovered before finer particles.^13^ For MPs, size and density are decoupled.^20^ Also, MPs are generally less dense than natural particles, with density varying between 0.8 and 1.34 g/cm^3^ in comparison to over 2.0 g/cm^3^ for natural colloids.^16^

Li et al. explored how MP density and particle size in relation to aquifer grain size can affect MP transport and retention in porous media.^79,80^ The results from column experiments suggest that, for the same size class (27–35 μm), MP particles with density similar to or lower than water density are more mobile than MP particles denser than water, regardless of the material of the media (glass beads or natural gravel).^79^ In a second column experiment using PE microspheres with a density close to 1 g/cm^3^ and size varying from 10 to 150 μm, results point to an increased mobility of MP with increased sediment size and decreased particle size.^80^

A column experiment performed by Rieckhof et al. investigated how transport of PS fluorescent microspheres (<10 μm) is affected by particle size through quartz sand under unsaturated conditions. They observed that larger particles (>1 μm) tend to get trapped in small pores due to straining. However, when they are not trapped, larger particles tend to travel faster through preferential flows due to the size-exclusion effect, arriving earlier at the column outlet than dissolved tracers.^81^ Experiments performed by Schenkel et al. showed MP retardation in comparison with NaCl tracer; however, they were inconclusive to what extent polymer type and particle shape affect MP retardation and retention.^82^ As particulate tracers, MP could inform about preferential flow, effective permeability, and bypass flow in porous aquifers. Because of filtration, retention, and retardation effects in porous media, MP in these settings would most likely reflect local sources of contamination.

Once MP particles reach karstic and fractured aquifers, physical filtration is minimal, and particles can travel long distances without attenuation. (2024) observed how the size and shape of MP particles recovered from a fractured aquifer in Korea were affected by the volume of the sample. They noted that smaller particles and fragments are removed before larger particles and fibers.^65^ In karst systems with a rapid conduit response to precipitation, MP pulses following storms can help delineate recharge dynamics. The scale of investigations in these aquifer types ranges from the catchment to the regional scale.

When analyzing the specific polymer type of individual MPs present in groundwater in areas where natural recharge processes are predominant, it is possible to use the year of polymer creation to set the maximum infiltration date. If the groundwater age was measured by other methods and it was older than the polymers found, it may indicate groundwater mixing or sample contamination. Being a pollutant that appeared less than a hundred years ago, MPs have not yet had sufficient time to integrate into the deep and regional groundwater flow, which could take millennia. In the case of MPs being found in older/deeper groundwater, it could be the result of MPs being directly introduced in deeper layers of the aquifer by artificial processes such as managed aquifer recharge (MAR) via deep boreholes, pipe leakage, or drilling and excavation activities.^77^

The relationship between land use and MP polymer composition and abundance found in groundwater is already established,^19,56,63^ providing insights into potential pollution sources. For example, recent reviews reported that agricultural areas were characterized by less variability in MP polymer composition due to specialization of plastic use in their activities, with PP and PE being the predominant types, whereas urban settings show the highest variability in polymer types.^19,56^ Depending on the scale of the study and the hydraulic conductivity of the aquifer, it is possible that variable land use in the hydrogeological basin could affect the MP characteristics at the sampling point.^19^ Samandra et al., investigating MP contamination in a shallow unconfined aquifer in Victoria, Australia, were able to combine polymer type with local land use information to identify potential MP pollution sources.^63^

A review paper from He et al., after comparing data from 76 studies, found that areas with high anthropogenic impact such as landfills, agricultural, urban, and industrial areas were identified as areas with high MP concentrations in groundwater.^19^ The highest concentrations were recorded in association with landfills, being 3 to 6 times higher than those in industrial and residential areas. In agricultural areas, MP concentration showed a big internal variation, with a median value of 1.0 particles per liter. In contrast, low MP concentrations were described in areas with no or minimal anthropogenic activity. In those areas, MP occurrence could be related to atmospheric transport and deposition and/or fluvial transport and exchange with groundwater.^19,56^

The relationship observed between land use around the sampling area and polymer type found in groundwater could be associated with MP circulation and residence time in the aquifers. This corroborates the hypothesis that, without major artificial influences on recharge processes, MP pollution in groundwater reflects the local groundwater circulation, therefore reflecting local sources of pollution (Figure 2).

**Figure 2:**
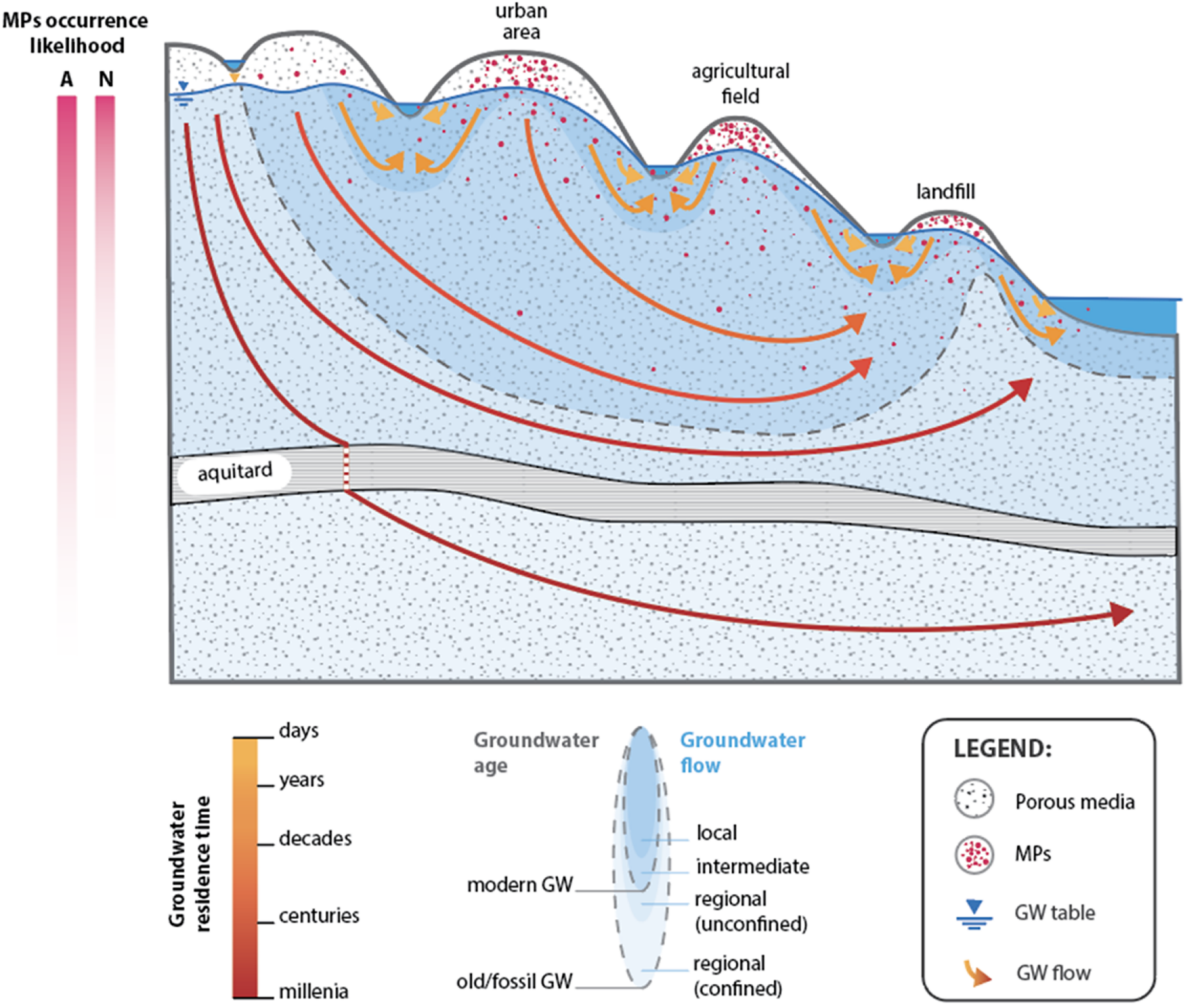
MPs occurrence in groundwater (GW) in relation to GW flow and age, and their association with local sources of pollution. N’ MPs refers to the particles present in sediments and soils due to contaminated surface waters and runoff, agricultural activities, plastic waste mismanagement, and atmospheric deposition. They are expected to be found in modern groundwater, associated with local and intermediate GW flows, and would reflect local sources of pollution. A’ MPs refers to the particles inserted directly into GW by MAR, leakage of pipes, and drilling activities. They could reach deeper levels of the aquifer, getting integrated into the regional flows and contaminating old GWs. Concentration of N’ ≫ A’ MPs.

Some tracers that could be used in the multitracer investigation are listed in Table 1, along with their application, residence time covered, costs, strengths, and limitations. The choice of cotracers can be influenced by a range of hydrogeological, chemical, environmental, and financial factors. Antibiotics, pharmaceuticals, and pesticides are useful indicators for distinguishing contamination sources (e.g., agricultural and sewage), which can often be linked to current or past land use. Because the degradation rates of these compounds can vary from days to years, depending on their chemical structure and environmental conditions, the selection of target compounds should be guided by preliminary geochemical and hydrogeological characterization. Surface chemical properties of the aquifer can affect the occurrence, degradation rate, and mobility of these contaminants. For instance, hydrophilic contaminants can be adsorbed onto the mineral surface and have their mobility reduced whenever clay minerals are present in the aquifer.

**Table 1:** Summary of Commonly Used and Novel Groundwater Tracers, Their Applications, Age Interval Covered, Costs, Strengths, Limitations, and Potential for Combined use with MPs^a^

**tracer**	**application**	**age interval covered**	**cost**	**strength**	**limitation**	**potential for joint use with MPs**
CECs (antibiotics, pharmaceuticals, pesticides, additives)	urban/peri-urban areas Agricultural zones	days/months/years	$	detailed information on contamination sources and useful applicability for residence time assessment.	multiple sources, very prone to cross-contamination	cotracers for processes, sources, and residence time
CFCs	rural and semipristine settings	years (CFC-11 and CFC-12 from 1950s to present and CFC-113 from 1970s to present)	$$$	patterns among multiple CFCs show water mixing and contamination	contaminated areas restrict the application of the method (e.g., urban areas)	cotracers for sources
δ^3^H/^3^He	urban or agricultural areas, tracer tests	years	$$–$$$	ideal for tracing recent contamination or recharge events	few laboratories perform this analysis	cotracers for processes and sources
δ^85^Kr	urban and rural areas, and semipristine settings	years (post-1950s recharge)	$$$$	useful for tracing contamination, leakage, or water supply dynamics	requires very-sensitive equipment; very limited laboratories needs groundwater without turbidity	ideal for intermediate ages, cotracer for dating
SF_6_	urban/peri-urban areas agricultural zones	years (post-1950s recharge)	$$$	low-cost, practical tracer for routine groundwater studies; best for young, shallow, or mixed systems	contaminated areas restrict the application of the method	complements CFCs to better define groundwater age^87^
δ^14^C	urban/peri-urban areas Agricultural zones	up to ∼50,000 years	$$$	standardized application	great volume of sample needed for analyses	cotracers for dating

a $: 100–200 euros; $$:200–500 euros; $$$: 500–1000 euros; $$$$ > 1000 euros.

Radiocarbon (δ^14^C) is an effective and relatively simple dating method in terms of sampling requirements; however, it is generally applied to date groundwater in the range of 1000 to 40,000 years.^33^ The detection of MPs in such old waters, whenever other types of contamination during sampling and processing can be neglected, could therefore indicate cross-contamination through well structures, MAR through deep boreholes, drilling, and excavation activities.

Sampling protocols for CFCs are straightforward, and the results provide good age resolution. The main limitation of this tracer is its application in areas where widespread CFC contamination masks the environmental background concentration.^32^ This is the case in most urban areas, where high MPs concentrations are expected. Similarly, SF_6_ cannot be used in areas affected by local industrial contamination.^77,83^ To overcome such contamination issues, alternative dating methods such as δ^85^Kr and δ^3^H/^3^He can be employed. These tracers provide excellent results even for very young groundwater, however complex sampling procedures are required and only a limited number of laboratories are capable of performing the analyses.^84^

For δ^85^Kr, the dissolved gas must be separated from the water matrix in situ and collected in a sealed container for laboratory analysis. The gas–water separation is typically achieved using a membrane extractor, which can become clogged or damaged by suspended material, potentially compromising sample integrity. This makes δ^85^Kr sampling particularly challenging in shallow aquifers, where elevated turbidity and high MP concentrations are expected.^85^ The δ^3^H/^3^He tracer method can be applied to very young waters (<50 years), but it requires a technically demanding sampling procedure, involving the collection of water in gastight copper tubes by trained personnel.^84,86^ Additional limitations include the potential for cross-contamination with atmospheric gases during sampling, degassing during pumping, and reduced reliability in aquifer systems characterized by mixing.

Integrating MPs as cotracers alongside novel and conventional groundwater age tracers can provide complementary insights into both contamination sources and aquifer dynamics. MPs are predominantly modern anthropogenic pollutants, and their occurrence in groundwater can serve as a qualitative indicator of recent recharge, helping to corroborate ages inferred by tracers such as CFCs, SF_6_, or δ^3^H/^3^He. Beyond age, MPs carry source-specific information (e.g., polymer type, size, and additive composition) that can reveal land use contributions, distinguishing between industrial, agricultural, landfill, and sewage-derived inputs.

The transport and retention of MPs are strongly influenced by aquifer properties, such as effective porosity and clay content, which control preferential flow paths, filtration effects, and delayed contaminant transport. Beyond these physical properties, geochemical conditions within the aquifer can further influence particulate behavior. In particular, redox conditions may alter particle surface charge, aggregation behavior, and interactions with mineral surfaces, thus affecting MP mobility and retention in porous media. Evidence from colloid studies shows that redox processes can significantly influence particle transport behavior in such media.^88,89^ By providing an independent record of modern anthropogenic influence, MPs can also serve as a cross-validation tool for conventional tracers, highlighting potential mixing, well cross-contamination, or anomalous apparent ages. Together, MPs and chemical age tracers form a complementary framework that enhances our understanding of both groundwater residence times and the pathways of recent contamination in complex aquifer systems.

### Sampling and Measuring MPs in Groundwater

Although there is scientific evidence of the presence of MPs in groundwater, there is currently no globally accepted standardized analytical method for MP sampling, extraction, and analysis in groundwater.^90^ Currently, the lack of standardized methods for sampling and measuring MPs makes it challenging to compare results from different studies, and the lack of consistency in measuring and reporting results means that, on occasion, it is not clear what is being measured.^22–24,95^ The most common analytical techniques, outputs, and limitations are summarized in Table 2.

**Table 2:** Summary of Common Analytical Techniques to Characterize MPs Based on a Three-Tier MP Characterization Approach Proposed by Coffin^91^

**method**	**outputs**	**advantages**	**limitations**	**example**
total particle analysis (screening method)	assess potential MP abundance. Identify the number of particles in a sample extract. It is often the first step in estimating MP concentration.	cost-efficient, estimate the upper thresholds of MP concentration.	can overestimate MP concentration (also quantifies inorganic and organic suspended solids and nonplastic particles)	light scattering, visual spectroscopy, microscopy (optical, electron, fluorescence)
total MP methods	bulk quantification of MPs by mass. Identify whether particles are composed of plastics or not.	exclude inorganic or nonplastic particles. More cost-efficient than chemical identification methods. Provide information on the MP polymer type and additives.	analyses one particle at a time. Do not give information on particle size or shape distribution.	pyrolysis gas chromatography mass-spectrometry (Py-GCMS)
chemical identification	identify the chemical fingerprints of individual particles via spectral analysis.	can provide information on polymer composition, size, and shape. Some techniques can also provide mass-based concentration. These characteristics can hint about possible sources and help estimating environmental risk.	time and labor-intensive. Expensive compared to other methods. Requires extensive training in data processing.	Fourier transformed infrared (FTIR), Raman spectroscopy, laser direct infrared (LDIR), near infrared imaging spectroscopy (NIR), scanning electron microscopy with energy dispersive X-ray spectroscopy (SEM-EDS, SEM-EDX)

Different analytical pathways led to different results. Recent literature reviews and prospects^19,56^ describe how variable lower detection limits for MP particle size between different methods could affect the quantification of MP particles in a sample. Additionally, black particles are generally challenging to identify via fluorescence microscopy or standard optical methods such as FTIR or Raman spectroscopy, which may result in underestimation of MP abundance, in particular considering the unaccounted-for burden of dark tire and road wear particles.

When sampling groundwater from a well, whether it has been purged or not, and the extent of purging, affect the quantity of MPs identified in samples.^56,63,92^ Wells that have not been purged before sampling show MP concentrations up to 3 orders of magnitude higher than pumped samples.^92^ Cha et al. observed on their study site that MP concentration decreases as the sampling volume increases, with lighter and smaller fragments being removed before heavier and larger particles and long fibers.^65^

For characterizing MP occurrence in groundwater, an important first step is defining a suitable sampling protocol, including sample volume, based on the characteristics of the aquifer in the study area (Figure 3). An early study by Koelmans et al.^93^ recommended the sampling of a minimum of 500 L for river waters, regardless of the chosen sampling approach, due to the low concentrations of MPs. Rohais et al.^25^ suggest that this approach should be extended to different environments to facilitate comparison among measurements. Kim et al.,^74^ based on a study in a very productive fractured basaltic aquifer in South Korea, also recommended 500 L as the minimum amount for a representative GW sample.

**Figure 3:**
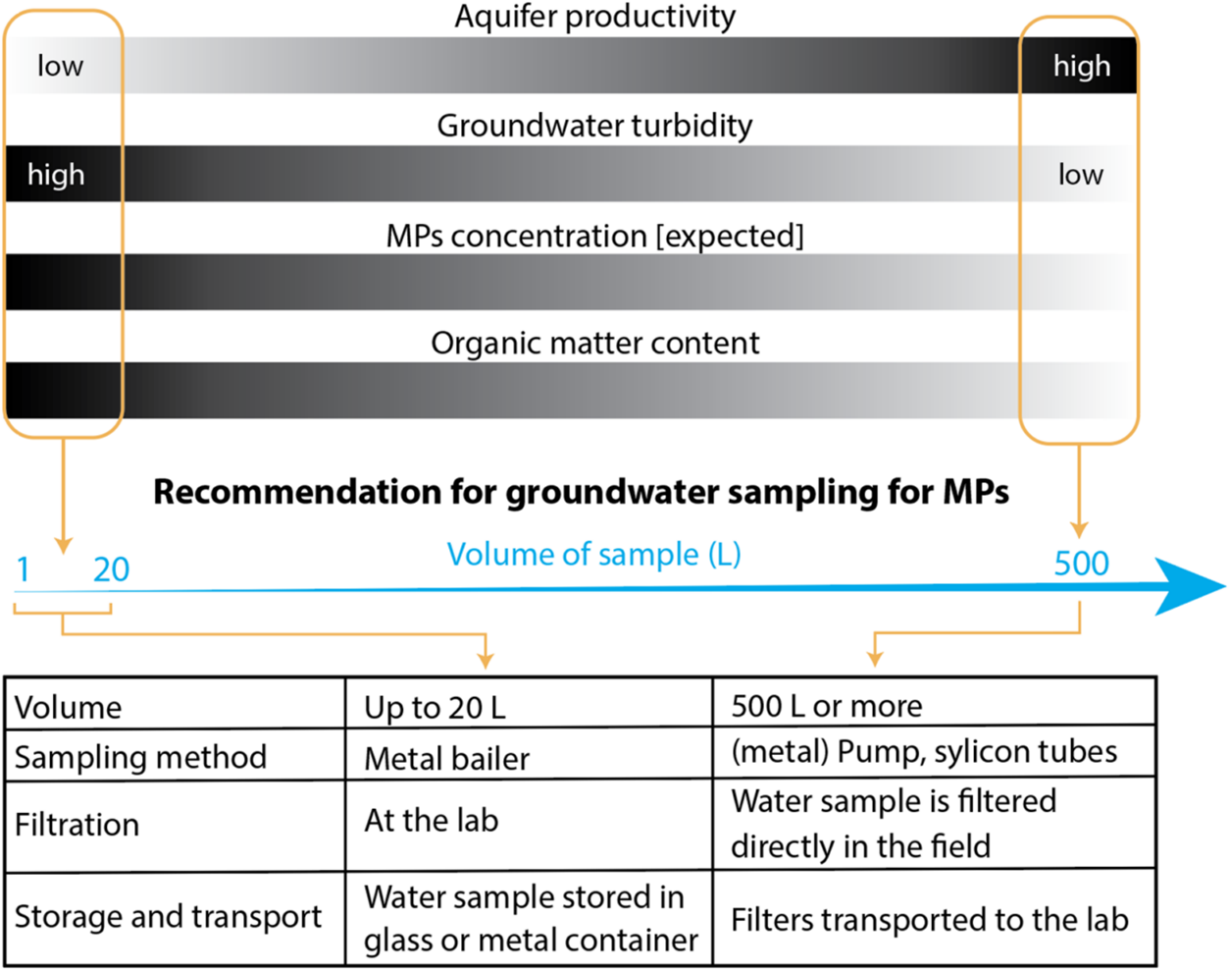
Recommendation for groundwater sampling for MPs based on the characteristics of the aquifer and the groundwater.

Typically, for small volumes of groundwater—up to 20 L, samples are stored in glass or stainless-steel containers and transported to the laboratory for MPs extraction and separation.^64,68,78,79^ This is frequently the case for high turbidity, high organic matter content, low aquifer productivity, and when high amounts of MPs are expected to be found. When groundwater turbidity and organic matter content are low, and also low MP concentrations are expected, higher volumes of groundwater should be sampled. In this case, groundwater should be filtered directly in the field and the filters should be transported to the laboratory.^54,92,94^ Some of the challenges and limitations of working with MPs in groundwater are listed in Box [Boxed-text box1].

1 **Current challenges and limitations of working with MPs in groundwater**

- Large groundwater volume requirements are impractical for low-productivity aquifers or wells
- Transport of large volumes of water to the laboratory is logistically challenging
- If sampling is done with a bailer, field filtration could introduce cross contamination risks (such as atmospheric deposition). That would not be the case for closed filters or filter cascades attached to pumping systems
- Trade-off between sample representativeness and analytical feasibility
- Context-dependent approaches for sampling should be prioritised
- Quality control and assurance remain critical challenges

In most publications results are presented as a particle concentration (particles per liter), whereas others report concentration by mass (grams per liter). The former (particles per liter) is useful to understand MPs transport inside the aquifer, since the density varies for each polymer, the size and shape of the particle affect the way it flows inside the aquifer,^79,80^ and the surface area and level of weathering affect its chemical bonds.^95^ The latter (grams per liter) represents the actual concentration of plastics in water and should be used to define exposure limits in regulatory measures. To overcome the challenge posed by the lack of standardization would be to promote open data reporting on full MP data sets, including the type of aquifer sampled, depth of the boreholes, volume of samples, whether the borehole was purged or not, polymer types, size classes, shape and color when available, equipment detection limits, and thresholds used. In this way, even with methodological constraints, data sets could still be compared for overlapping hydrogeological settings, polymer groups, or size fractions. That would not be the case for closed filters or filter cascades attached to pumping systems

### Quality Assurance and Quality Control during MP Analysis

When measuring MPs in groundwater or in any other environmental medium, avoidance or limitation of contamination, as well as ensuring reliability and reproducibility of results, remains a challenge. Figure 4 summarizes approaches employed by researchers to generate reliable data and avoid over- or underestimation of MP concentrations.^19,96,97^ All measures combined will increase the reliability of results and reduce the risk of contamination, especially in the field, where cross-contamination is sometimes unavoidable.

**Figure 4:**
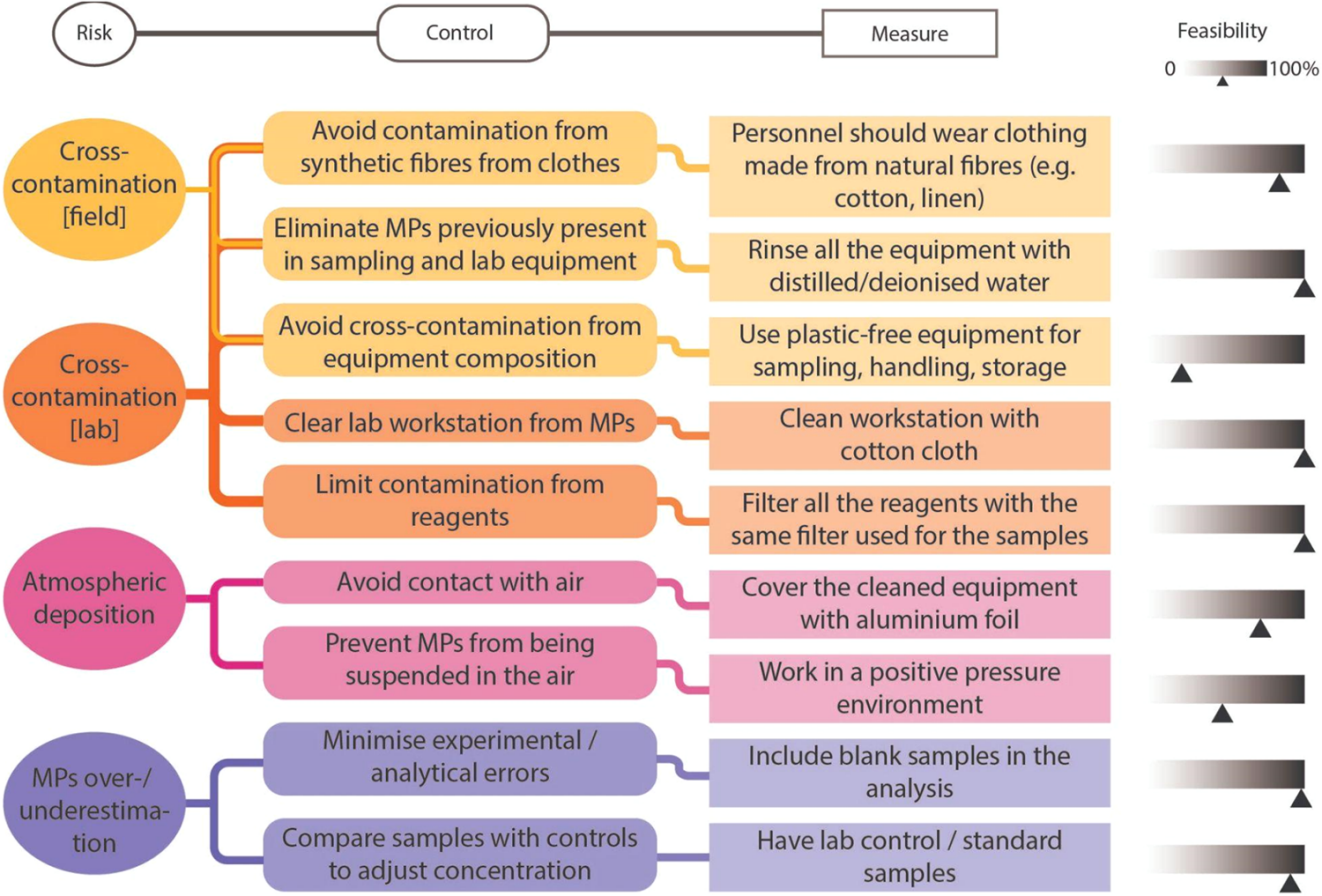
Common QA/QC methods.^19,96,97^

The use of positive and negative controls, including field and laboratory blanks when needed, further increases the reliability of the results. A negative control involves collecting and analyzing blank samples in the field and in the lab to identify and quantify any contamination occurring during sampling and analysis. In case an open sampling approach was chosen due to low aquifer productivity, high groundwater turbidity, and/or organic matter content, a field blank would be useful to account for atmospheric deposition. For large volumes of groundwater, when samples are typically collected using pumps and in-line filtration systems, blanks can be designed to assess potential contamination from pumps, well materials, tubing, or filters. Positive controls refer to the use of control/standard MP samples in the lab to compare with the study findings to avoid misidentification of MPs.

There are a few advantages of working with groundwater in comparison to those of other aquatic matrices. Because groundwater systems are much more shielded compared with surface waters, if a closed sampling loop is maintained, the risk of atmospheric contamination during sampling can be kept minimal. Also, surface water systems change faster over time; thus, temporal variability between groundwater samples is expected to be lower, meaning there is more insight to be gained even from low sample numbers as long as they are reliable.

### Other Challenges and Limitations

When researching MPs pollution in groundwater, some practical limitations must be acknowledged. Residence time studies often rely on a small volume of isotope samples, usually a few mL, whereas microplastics require large volume sampling, ranging from a few liters to hundreds of liters. Another limitation of MP application as tracers in comparison with isotopes is the temporal resolution covered. While for MP, the signal is restricted to “post-1950s,” isotopes can capture both modern and ancient recharge history.

Some additional layers of complexity were expected. MPs can both interact with other contaminants present in aqueous media via adsorption, cotransportation, and chemical processes and leach their additives to the environment due to weathering processes. These interactions can influence MP distribution, fate, environmental toxicity, and potential bioaccumulation.^96,98,99^ These factors have to be taken into consideration when interpreting the results obtained from MP analysis in a study area in combination with other tracers.

Being mostly hydrophobic substances with a large surface area, MPs serve as carriers for other hydrophobic chemicals and metal ions. MPs can carry and potentially interact with chemicals such as antibiotics, preservatives, UV filters, β-blockers, per- and polyfluoroalkyl substances (PFAS), bisphenol A (BPA), polychlorinated biphenyls (PCBs), polycyclic aromatic hydrocarbons (PAHs), and flame retardants.^96,99^ The MP adsorption capacity is influenced by the chemical nature of the polymer functional groups and polarity.^100^ The increased surface area of aged MPs facilitates the physical attachment of other substances. Leaching of physically bonded chemicals depends on temperature, pH, and light exposure.^98^ With regard to groundwater systems where there is no UV exposure, there is still no information available on how MPs weather.

From a chemical perspective, plastic additives added intentionally during manufacture, such as plasticizers, stabilizers, flame retardants, and other chemicals, will leach from the plastic into the environment during use and following disposal at end-of-life. Different chemicals are associated with different polymer types,^98^ thus the leaching of chemicals from MPs relies heavily on the polymer type, the physicochemical nature of the additive, and how it is incorporated into the polymer. Factors affecting MP chemical leaching in aqueous media include MP concentration, polymer, time span, pH, and contact time.^98^

## Conclusion and Outlook

This study explores the potential of MPs to act as groundwater environmental cotracers and to outline the methodological and conceptual advances required to enable their effective integration into groundwater research. The findings presented here support the plausibility of using MPs within a multitracer framework to improve the robustness of hydrogeological conceptual models and to refine our understanding of subsurface contamination processes. When interpreted jointly with established tracers such as water-stable isotopes, major and trace elements, CECs, and physicochemical parameters, MPs may contribute an additional dimension of information, particularly regarding particulate transport, recharge time scale, and anthropogenic influence.

The use of MPs as cotracers is particularly relevant in young/modern groundwater systems, which constitute the active component of the hydrological cycle and supply most of the world’s drinking water. These systems are often more directly connected to surface environments, making them especially susceptible to anthropogenic inputs. Given that plastic production expanded globally only after the 1950s, MPs detected in groundwater may serve as event markers delineating recent recharge or contamination episodes. Conversely, the presence of MPs in aquifers containing water older than the polymers’ creation date may indicate groundwater mixing or contamination through preferential pathways or artificial recharge systems. Such insights have direct implications for understanding subsurface connectivity and for identifying zones of increased vulnerability to contamination.

Despite these promising perspectives, several limitations constrain the current application of MPs as cotracers. Field contamination during sampling and laboratory handling further threatens data integrity, necessitating stringent quality assurance and control procedures. Moreover, interpreting MP signals in areas subjected to artificial recharge or strong anthropogenic modification requires caution, since such systems may obscure natural groundwater flow and mixing patterns. Interactions between MPs and co-occurring contaminants resulting in adsorption, cotransport, and additive leaching may further modify their fate, transport, and toxicity in ways that are not yet fully understood. Finally, the analytical techniques required for MP characterization remain costly and labor-intensive, posing financial barriers to their widespread adoption in groundwater monitoring and limiting their routine use, particularly in resource-constrained contexts.

To facilitate the reliable use of MPs as groundwater cotracers, the development of context-specific sampling strategies adapted to local hydrogeological settings is of greater benefit than the pursuit of a universal protocol. Emphasis should be placed on integrating MP data with hydrochemical and isotopic data sets to enhance interpretability and cross-validation and promoting open data reporting on full MP data sets. Future research should focus on (i) elucidating how particle size, polymer type, and surface aging affect MPs transport and retention in field settings; (ii) extending investigations to older and deeper aquifers to assess long-term migration potential and effects of artificial recharge in groundwater pollution; (iii) designing remediation approaches for aquifers affected by plastic pollution; and (iv) addressing the logistical and financial challenges associated with MPs analyses through collaborative infrastructure, training, and data-sharing initiatives. Such interdisciplinary efforts will be essential to overcoming current technical and conceptual barriers.

In conclusion, this work demonstrates the potential for the use of MPs as a novel class of environmental cotracers for groundwater studies. Their integration into multitracer frameworks could advance the capacity to understand groundwater recharge dynamics, MP contamination sources, and groundwater mixing. Although methodological, financial, and interpretative challenges remain, incorporating MPs into the broader groundwater tracing toolkit offers a promising path toward a more comprehensive understanding of anthropogenic impacts on the subsurface environment and toward more informed strategies for sustainable groundwater management.

## Supplementary Material



## Notes

Figure 1: Tables with the date of discovery and commercialization of polymers, first reported use, global polymer production by decade, and key references and data sources (PDF)

## References

[1] Thompson R. C., Swan S. H., Moore C. J., Vom Saal F. S. (2009). Our Plastic Age. Philos. Trans. R. Soc., B.

[2] Plastics Europe Plastics-the Facts 2023 -an Analysis of European Plastics Production, Demand and Waste Data; Plastics Europe, 2023; https://plasticseurope.org/knowledge-hub/plastics-the-fast-facts-2023/. (accessed Nov 15, 2024).

[3] Okoffo E. D., Donner E., McGrath S. P., Tscharke B. J., O’Brien J. W., O’Brien S., Ribeiro F., Burrows S. D., Toapanta T., Rauert C., Samanipour S., Mueller J. F., Thomas K. V. (2021). Plastics
in Biosolids from 1950 to 2016: A Function of Global Plastic Production
and Consumption. Water Res..

[4] Dokl M., Copot A., Krajnc D., Fan Y. V., Vujanović A., Aviso K. B., Tan R. R., Kravanja Z., Čuček L. (2024). Global Projections
of Plastic Use, End-of-Life Fate and Potential Changes in Consumption,
Reduction, Recycling and Replacement with Bioplastics to 2050. Sustainable Prod. Consumption.

[5] Kaza, S. ; Yao, L. What a Waste 2.0: A Global Snapshot of Solid Waste Management to 2050; Urban development series; World Bank Group: WA, 2018.

[6] Crawford, C. B. ; Quinn, B. Microplastic Pollutants; Elsevier: Amsterdam, 2017.

[7] Thompson R.
C., Courtene-Jones W., Boucher J., Pahl S., Raubenheimer K., Koelmans A. A. (2024). Twenty Years of Microplastic Pollution Research—What
Have We Learned?. Science.

[8] Xayachak T., Haque N., Lau D., Pramanik B. K. (2024). The Missing Link:
A Systematic Review of Microplastics and Its Neglected Role in Life-Cycle
Assessment. Sci. Total Environ..

[9] Groundwater Making the Invisible Visible, UN Water, Ed.The United Nations world water development report; UNESCO: Paris, 2022.

[10] Currell M., McCance W., Jones O. A. H. (2022). Novel Molecular
Tracers for the Assessment
of Groundwater Pollution. Curr. Opin. Environ.
Sci. Health.

[11] Currell M. J., Han D. (2017). The Global Drain: Why China’s Water Pollution Problems Should
Matter to the Rest of the World. Environ. Sci.
Policy Sustainable Dev..

[12] Freeze, R. A. ; Cherry, J. A. Groundwater; Prentice-Hall: Englewood Cliffs, N.J, 1979.

[13] Massei N., Lacroix M., Wang H. Q., Dupont J.-P. (2002). Transport of Particulate
Material and Dissolved Tracer in a Highly Permeable Porous Medium:
Comparison of the Transfer Parameters. J. Contam.
Hydrol..

[14] Harvey R. W. (1997). Microorganisms
as Tracers in Groundwater Injection and Recovery Experiments: A Review. FEMS Microbiol. Rev..

[15] Burkhardt M., Kasteel R., Vanderborght J., Vereecken H. (2008). Field Study
on Colloid Transport Using Fluorescent Microspheres. Eur. J. Soil Sci..

[16] Schiperski F., Zirlewagen J., Scheytt T. (2016). Transport and Attenuation of Particles
of Different Density and Surface Charge: A Karst Aquifer Field Study. Environ. Sci. Technol..

[17] Vucinic L., O’Connell D., Coxon C., Gill L. (2024). Back to the Future:
Comparing Yeast as an Outmoded Artificial Tracer for Simulating Microbial
Transport in Karst Aquifer Systems to More Modern Approaches. Environ. Pollut..

[18] Goeppert N., Goldscheider N. (2019). Improved Understanding of Particle
Transport in Karst
Groundwater Using Natural Sediments as Tracers. Water Res..

[19] He Y.-Q., McDonough L. K., Zainab S. M., Guo Z.-F., Chen C., Xu Y.-Y. (2024). Microplastic
Accumulation in Groundwater: Data-Scaled Insights and
Future Research. Water Res..

[20] Koelmans A. A., Redondo-Hasselerharm P. E., Nor N. H. M., De
Ruijter V. N., Mintenig S. M., Kooi M. (2022). Risk Assessment of
Microplastic Particles. Nat. Rev. Mater..

[21] Kitahara K.-I., Nakata H. (2020). Plastic Additives as Tracers of Microplastic Sources
in Japanese Road Dusts. Sci. Total Environ..

[22] Di
Bella G., Albergamo A., Litrenta F., Lo Turco V., Potortì A. G. (2024). Can Phthalates Be Considered as Microplastic Tracers
in the Mediterranean Marine Environment?. Environments.

[23] Pittroff M., Loui C., Oswald S. E., Bochow M., Kamp J., Dierkes G., Lensing H.-J., Munz M. (2024). Riverbed Depth-Specific
Microplastics Distribution and Potential Use as Process Marker. Environ. Sci. Pollut. Res..

[24] Van
Sebille E., Aliani S., Law K. L., Maximenko N., Alsina J. M., Bagaev A., Bergmann M., Chapron B., Chubarenko I., Cózar A., Delandmeter P., Egger M., Fox-Kemper B., Garaba S. P., Goddijn-Murphy L., Hardesty B. D., Hoffman M. J., Isobe A., Jongedijk C. E., Kaandorp M. L. A., Khatmullina L., Koelmans A. A., Kukulka T., Laufkötter C., Lebreton L., Lobelle D., Maes C., Martinez-Vicente V., Morales Maqueda M. A., Poulain-Zarcos M., Rodríguez E., Ryan P. G., Shanks A. L., Shim W. J., Suaria G., Thiel M., Van Den Bremer T. S., Wichmann D. (2020). The Physical Oceanography of the Transport of Floating
Marine Debris. Environ. Res. Lett..

[25] Rohais S., Armitage J. J., Romero-Sarmiento M.-F., Pierson J.-L., Teles V., Bauer D., Cassar C., Sebag D., Klopffer M.-H., Pelerin M. (2024). A Source-to-Sink Perspective
of an Anthropogenic Marker:
A First Assessment of Microplastics Concentration, Pathways, and Accumulation
across the Environment. Earth-Sci. Rev..

[26] Drummond J. D., Nel H. A., Packman A. I., Krause S. (2020). Significance of Hyporheic
Exchange for Predicting Microplastic Fate in Rivers. Environ. Sci. Technol. Lett..

[27] Drummond J. D., Schneidewind U., Li A., Hoellein T. J., Krause S., Packman A. I. (2022). Microplastic Accumulation
in Riverbed Sediment via
Hyporheic Exchange from Headwaters to Mainstems. Sci. Adv..

[28] Loui C., Pittroff M., Oswald S. E., Straßer D., Bochow M., Munz M. (2026). Polymer-Specific Transfer and Retention
of Microplastics at the River–Sediment–Groundwater Interface. Water Res..

[29] Goeppert N., Goldscheider N. (2021). Experimental Field Evidence for Transport
of Microplastic
Tracers over Large Distances in an Alluvial Aquifer. J. Hazard. Mater..

[30] Harvey, R. W. ; Harms, H. Tracers in Groundwater: Use of Microorganisms and Microspheres. In Encyclopedia of Environmental Microbiology; Bitton, G. , Ed.; Wiley, 2003; Vol. 6, pp 3194–3202 10.1002/0471263397.env157.

[31] Ward, R. S. ; Harrison, I. ; Leader, R. U. ; Williams, A. T. Fluorescent Polystyrene Microspheres as Tracers of Colloidal and Particulate Materials: Examples of Their Use and Developments in Analytical Technique. In Tracer Hydrology 97; Kranjc, A. , Ed.; CRC Press, 2020; pp 99–103 10.1201/9781003078142-18.

[32] Evans G. V. (1983). Tracer
Techniques in Hydrology. Int. J. Appl. Radiat.
Isot..

[33] Environmental Tracers in Subsurface Hydrology, Cook, P. G. ; Herczeg, A. L. , Eds.; Springer Science+Business Media, LLC: New York, 2000.

[34] Suckow A. (2014). The Age of
Groundwater – Definitions, Models and Why We Do Not Need This
Term. Appl. Geochem..

[35] Cook, P. Introduction to Isotopes and Environmental Tracers as Indicators of Groundwater Flow; The Groundwater Project, 2020. 10.21083/978-1-7770541-8-2.

[36] Davis S. N., Thompson G. M., Bentley H. W., Stiles G. (1980). Ground-Water Tracers
— A Short Review. Groundwater.

[37] Clark, I. D. ; Fritz, P. Environmental Isotopes in Hydrogeology, 1st ed.; CRC Press, 2013 10.1201/9781482242911.

[38] Gardner W. P., Harrington G. A., Solomon D. K., Cook P. G. (2011). Using Terrigenic^4^ He to
Identify and Quantify Regional Groundwater Discharge
to Streams. Water Resour. Res..

[39] Sukanya S., Noble J., Joseph S. (2022). Application
of Radon (222Rn) as an
Environmental Tracer in Hydrogeological and Geological Investigations:
An Overview. Chemosphere.

[40] Lindsey, B. ; Jurgens, B. ; Bellitz, K. Tritium as an Indicator of Modern, Mixed, and Premodern Groundwater Age; Scientific Investigations Report; Report;U.S. Geological Survey:: Reston VA, 2019; 10.3133/sir20195090.

[41] Solomon D.
K., Sudicky E. A. (1991). Tritium
and Helium 3 Isotope Ratios for Direct Estimation
of Spatial Variations in Groundwater Recharge. Water Resour. Res..

[42] Darling W. G., Gooddy D. C., MacDonald A. M., Morris B. L. (2012). The Practicalities
of Using CFCs and SF6 for Groundwater Dating and Tracing. Appl. Geochem..

[43] Schubert M., Lin M., Clark J. F., Kralik M., Damatto S., Copia L., Terzer-Wassmuth S., Harjung A. (2024). Short-Lived Natural Radionuclides
as Tracers in Hydrogeological Studies – A Review. Sci. Total Environ..

[44] Widory D., Kloppmann W., Chery L., Bonnin J., Rochdi H., Guinamant J.-L. (2004). Nitrate
in Groundwater: An Isotopic Multi-Tracer Approach. J. Contam. Hydrol..

[45] Pastén-Zapata E., Ledesma-Ruiz R., Harter T., Ramírez A. I., Mahlknecht J. (2014). Assessment
of Sources and Fate of Nitrate in Shallow
Groundwater of an Agricultural Area by Using a Multi-Tracer Approach. Sci. Total Environ..

[46] Filipović M., Terzić J., Lukač Reberski J., Vlahović I. (2024). Utilizing
a Multi-Tracer Method to Investigate Sulphate Contamination: Novel
Insights on Hydrogeochemical Characteristics of Groundwater in Intricate
Karst Systems. Groundwater Sustainable Dev..

[47] Wilske C., Suckow A., Mallast U., Meier C., Merchel S., Merkel B., Pavetich S., Rödiger T., Rugel G., Sachse A., Weise S. M., Siebert C. (2020). A Multi-Environmental
Tracer Study to Determine Groundwater Residence Times and Recharge
in a Structurally Complex Multi-Aquifer System. Hydrol. Earth Syst. Sci..

[48] Wilson J., Rocha C. (2016). A Combined Remote Sensing
and Multi-Tracer Approach for Localising
and Assessing Groundwater-Lake Interactions. Int. J. Appl. Earth Obs. Geoinformation.

[49] Balzani L., Orban P., Brouyère S. (2022). Protection
of Peri-Urban Groundwater
Catchments: A Multi-Tracer Approach for the Identification of Urban
Pollution Sources. Adv. Geosci..

[50] McCance W., Jones O. A. H., Edwards M., Surapaneni A., Chadalavada S., Currell M. (2018). Contaminants of Emerging
Concern
as Novel Groundwater Tracers for Delineating Wastewater Impacts in
Urban and Peri-Urban Areas. Water Res..

[51] McCance W., Jones O. A. H., Cendón D. I., Edwards M., Surapaneni A., Chadalavada S., Wang S., Currell M. (2020). Combining Environmental
Isotopes with Contaminants of Emerging Concern (CECs) to Characterise
Wastewater Derived Impacts on Groundwater Quality. Water Res..

[52] Richards L. A., Guo S., Lapworth D. J., White D., Civil W., Wilson G. J. L., Lu C., Kumar A., Ghosh A., Khamis K., Krause S., Polya D. A., Gooddy D. C. (2023). Emerging Organic
Contaminants in the River Ganga and Key Tributaries in the Middle
Gangetic Plain, India: Characterization, Distribution & Controls. Environ. Pollut..

[53] Crayol E., Huneau F., Garel E., Zuffianò L. E., Limoni P. P., Romanazzi A., Mattei A., Re V., Knoeller K., Polemio M. (2024). Investigating Pollution Input to
Coastal Groundwater-Dependent Ecosystems in Dry Mediterranean Agricultural
Regions. Sci. Total Environ..

[54] Mintenig S. M., Löder M. G. J., Primpke S., Gerdts G. (2019). Low Numbers of Microplastics
Detected in Drinking Water from Ground Water Sources. Sci. Total Environ..

[55] Re V. (2019). Shedding Light
on the Invisible: Addressing the Potential for Groundwater Contamination
by Plastic Microfibers. Hydrogeol. J..

[56] Lee J.-Y., Cha J., Ha K., Viaroli S. (2024). Microplastic Pollution in Groundwater:
A Systematic Review. Environ. Pollut. Bioavailability.

[57] Xu J., Zuo R., Wu G., Liu J., Liu J., Huang C., Wang Z. (2024). Global Distribution, Drivers, and
Potential Hazards of Microplastics
in Groundwater: A Review. Sci. Total Environ..

[58] Ganesan M., Nallathambi G., Srinivasalu S. (2019). Fate and Transport of Microplastics
from Water Sources. Curr. Sci..

[59] Li Y., Deng Y., Hu C., Li D., Zhang J., Zhou N. (2024). Microplastic Pollution in Urban Rivers
within China’s Danxia
Landforms: Spatial Distribution Characteristics, Migration, and Risk
Assessment. Sci. Total Environ..

[60] Alvarado-Zambrano D., Rivera-Hernández J. R., Green-Ruiz C. (2023). First Insight
into Microplastic Groundwater Pollution in Latin America: The Case
of a Coastal Aquifer in Northwest Mexico. Environ.
Sci. Pollut. Res..

[61] Patterson J., Laju R. L., Jeyasanta K. I., Shelciya S., Esmeralda V. G., Asir N. G. G., Narmatha M., Booth A. M. (2023). Hydrochemical Quality
and Microplastic Levels of the Groundwaters of Tuticorin, Southeast
Coast of India. Hydrogeol. J..

[62] S S., Subramani T., Prapanchan V. N., Peiyue L. (2023). Human Health Risk Perspective
Study on Characterization, Quantification and Spatial Distribution
of Microplastics in Surface Water, Groundwater and Coastal Sediments
of Thickly Populated Chennai Coast of South India. Hum. Ecol. Risk Assess. Int. J..

[63] Samandra S., Johnston J. M., Jaeger J. E., Symons B., Xie S., Currell M., Ellis A. V., Clarke B. O. (2022). Microplastic Contamination
of an Unconfined Groundwater Aquifer in Victoria, Australia. Sci. Total Environ..

[64] Mu H., Wang Y., Zhang H., Guo F., Li A., Zhang S., Liu S., Liu T. (2022). High Abundance
of Microplastics
in Groundwater in Jiaodong Peninsula, China. Sci. Total Environ..

[65] Cha J., Lee J.-Y., Lee J. (2024). Effects of
Groundwater Sample Volume
on Identified Microplastics in Groundwater of an Agricultural Area
in Korea. Sci. Total Environ..

[66] Severini E., Ducci L., Sutti A., Robottom S., Sutti S., Celico F. (2022). River–Groundwater
Interaction and Recharge Effects
on Microplastics Contamination of Groundwater in Confined Alluvial
Aquifers. Water.

[67] Esfandiari A., Abbasi S., Peely A. B., Mowla D., Ghanbarian M. A., Oleszczuk P., Turner A. (2022). Distribution and Transport of Microplastics
in Groundwater (Shiraz Aquifer, Southwest Iran). Water Res..

[68] Wan Y., Chen X., Liu Q., Hu H., Wu C., Xue Q. (2022). Informal Landfill Contributes
to the Pollution of Microplastics in
the Surrounding Environment. Environ. Pollut..

[69] Shu X., Xu L., Yang M., Qin Z., Zhang Q., Zhang L. (2023). Spatial Distribution
Characteristics and Migration of Microplastics in Surface Water, Groundwater
and Sediment in Karst Areas: The Case of Yulong River in Guilin, Southwest
China. Sci. Total Environ..

[70] Balestra V., Vigna B., De Costanzo S., Bellopede R. (2023). Preliminary
Investigations of Microplastic Pollution in Karst Systems, from Surface
Watercourses to Cave Waters. J. Contam. Hydrol..

[71] Panno S. V., Kelly W. R., Scott J., Zheng W., McNeish R. E., Holm N., Hoellein T. J., Baranski E. L. (2019). Microplastic Contamination
in Karst Groundwater Systems. Groundwater.

[72] Cha J., Lee J.-Y., Chia R. W. (2023). Microplastics
Contamination and Characteristics
of Agricultural Groundwater in Haean Basin of Korea. Sci. Total Environ..

[73] Gong X., Tian L., Wang P., Wang Z., Zeng L., Hu J. (2023). Microplastic Pollution
in the Groundwater under a Bedrock Island
in the South China Sea. Environ. Res..

[74] Kim Y.-I., Jeong E., Lee J.-Y., Chia R. W., Raza M. (2023). Microplastic
Contamination in Groundwater on a Volcanic Jeju Island of Korea. Environ. Res..

[75] Gleeson T., Befus K. M., Jasechko S., Luijendijk E., Cardenas M. B. (2016). The Global Volume and Distribution of Modern Groundwater. Nat. Geosci..

[76] Jasechko S., Perrone D., Befus K. M., Bayani Cardenas M., Ferguson G., Gleeson T., Luijendijk E., McDonnell J. J., Taylor R. G., Wada Y., Kirchner J. W. (2017). Global
Aquifers Dominated by Fossil Groundwaters but Wells Vulnerable to
Modern Contamination. Nat. Geosci..

[77] Viaroli S., Lancia M., Re V. (2022). Microplastics
Contamination of Groundwater:
Current Evidence and Future Perspectives. A Review. Sci. Total Environ..

[78] Perraki M., Skliros V., Mecaj P., Vasileiou E., Salmas C., Papanikolaou I., Stamatis G. (2024). Identification of Microplastics
Using Μ-Raman Spectroscopy in Surface and Groundwater Bodies
of SE Attica, Greece. Water.

[79] Li W., Brunetti G., Bolshakova A., Stumpp C. (2024). Effect of Particle
Density on Microplastics Transport in Artificial and Natural Porous
Media. Sci. Total Environ..

[80] Li W., Brunetti G., Zafiu C., Kunaschk M., Debreczeby M., Stumpp C. (2024). Experimental and Simulated
Microplastics Transport
in Saturated Natural Sediments: Impact of Grain Size and Particle
Size. J. Hazard. Mater..

[81] Rieckhof C., Martínez-Hernández V., Holzbecher E., Meffe R. (2024). Effect of Particle Size on the Transport
of Polystyrene Micro- and
Nanoplastic Particles through Quartz Sand under Unsaturated Conditions. Environ. Pollut..

[82] Schenkel C. A., Brown M. R. M., Lenczewski M. E. (2024). Impact
of Type and Shape of Microplastics
on the Transport in Column Experiments. Groundwater.

[83] Busenberg E., Plummer L. N. (2000). Dating
Young Groundwater with Sulfur Hexafluoride:
Natural and Anthropogenic Sources of Sulfur Hexafluoride. Water Resour. Res..

[84] Shapiro S. D., Rowe G., Schlosser P., Ludin A., Stute M. (1998). Tritium–Helium
3 Dating under Complex Conditions in Hydraulically Stressed Areas
of a Buried-valley Aquifer. Water Resour. Res..

[85] Zappala J.
C., Bailey K., Mueller P., O’Connor T. P., Purtschert R. (2017). Rapid Processing
of^85^ Kr/Kr Ratios Using
Atom Trap Trace Analysis. Water Resour. Res..

[86] Szabo Z., Rice D. E., Plummer L. N., Busenberg E., Drenkard S., Schlosser P. (1996). Age Dating of Shallow Groundwater
with Chlorofluorocarbons, Tritium/Helium: 3, and Flow Path Analysis,
Southern New Jersey Coastal Plain. Water Resour.
Res..

[87] Gooddy D. C., Darling W. G., Abesser C., Lapworth D. J. (2006). Using Chlorofluorocarbons
(CFCs) and Sulphur Hexafluoride (SF6) to Characterise Groundwater
Movement and Residence Time in a Lowland Chalk Catchment. J. Hydrol..

[88] Spielman-Sun E., Boye K., Dwivedi D., Engel M., Thompson A., Kumar N., Noël V. (2024). A Critical
Look at Colloid Generation,
Stability, and Transport in Redox-Dynamic Environments: Challenges
and Perspectives. ACS Earth Space Chem..

[89] Said-Pullicino D., Giannetta B., Demeglio B., Missong A., Gottselig N., Romani M., Bol R., Klumpp E., Celi L. (2021). Redox-Driven
Changes in Water-Dispersible Colloids and Their Role in Carbon Cycling
in Hydromorphic Soils. Geoderma.

[90] Jeon C., Kim H. (2024). Microplastics and Nanoplastics
in Groundwater: Occurrence, Analysis,
and Identification. Trends Environ. Anal. Chem..

[91] Coffin S. (2023). The Emergence
of Microplastics: Charting the Path from Research to Regulations. Environ. Sci. Adv..

[92] Munz M., Kreiß J., Krüger L., Schmidt L. K., Bochow M., Bednarz M., Bannick C. G., Oswald S. E. (2023). Application of High-Resolution
Near-Infrared Imaging Spectroscopy to Detect Microplastic Particles
in Different Environmental Compartments. Water.
Air. Soil Pollut..

[93] Koelmans A. A., Mohamed Nor N. H., Hermsen E., Kooi M., Mintenig S. M., De France J. (2019). Microplastics in Freshwaters and
Drinking Water: Critical
Review and Assessment of Data Quality. Water
Res..

[94] Jeong E., Kim Y.-I., Lee J.-Y., Raza M. (2023). Microplastic Contamination
in Groundwater of Rural Area, Eastern Part of Korea. Sci. Total Environ..

[95] Liu P., Zhan X., Wu X., Li J., Wang H., Gao S. (2020). Effect of Weathering on Environmental
Behavior of Microplastics:
Properties, Sorption and Potential Risks. Chemosphere.

[96] Nlend, B. ; Lapworth, D. ; Cashman, M. ; Lenczewski, M. Groundwater in the Age of Plastic. March 3, 2024 10.31223/X5QX3C.

[97] Viaroli S., Lancia M., Lee J.-Y., Ben Y., Giannecchini R., Castelvetro V., Petrini R., Zheng C., Re V. (2024). Limits, Challenges,
and Opportunities of Sampling Groundwater Wells with Plastic Casings
for Microplastic Investigations. Sci. Total
Environ..

[98] Haleem N., Kumar P., Zhang C., Jamal Y., Hua G., Yao B., Yang X. (2024). Microplastics and Associated Chemicals in Drinking
Water: A Review of Their Occurrence and Human Health Implications. Sci. Total Environ..

[99] Menéndez-Pedriza A., Jaumot J. (2020). Interaction
of Environmental Pollutants with Microplastics:
A Critical Review of Sorption Factors, Bioaccumulation and Ecotoxicological
Effects. Toxics.

[100] Fu L., Li J., Wang G., Luan Y., Dai W. (2021). Adsorption
Behavior of Organic Pollutants on Microplastics. Ecotoxicol. Environ. Saf..

