# Supplementary material for: Microplastics as Emerging Cotracers in Groundwater Quality Assessments
Source: ACS ES T Water. 2026 May 30;6(6):3300–13. doi: 10.1021/acsestwater.6c00063 (PMC13274488; doi:10.1021/acsestwater.6c00063)
Supplement: Supplementary file 1 [file ew6c00063_si_001.pdf]

Supplementary Information for the paper *Microplastics as emerging co-tracers in groundwater quality assessments* by Barbara Zambelli, Emma Pemberton, Stefano Viaroli, Stefan Krause, Roberto Giannecchini & Viviana Re

Table S 1: Polymer name, acronym, date of discovery and commercialization, first reported use

Polymer	Discovery / Commercialization	First Use
**PE (Polyethylene)**	1933 (LDPE)<br>1953 (HDPE)	1939 (ICI, UK)
**PP (Polypropylene)**	1954 (Natta/Ziegler)<br>1957 commercial	1957 (Montecatini, IT)
**PS (Polystyrene)**	1839 (natural)<br>1930s (synthetic)	1937 (BASF/Dow)
**PET (Polyethylene Terephthalate)**	1941 (Whinfield & Dickson, UK)	Late 1940s (fiber)<br>1970s (bottles)
**PVC (Polyvinyl Chloride)**	1872 (Baumann)<br>1926 (Waldo Semon)	1934 (Germany)<br>1937–38 (USA/Germany)
**PU (Polyurethane)**	1937 (O. Bayer, Germany)	1940s (coatings)<br>1952–54 (foams)
**PA (Polyamide / Nylon)**	1935 (Carothers/DuPont)<br>1938 commercial	1938–39 (DuPont, USA)
**PC (Polycarbonate)**	1953 (Schnell/Bayer & Fox/GE)	1958 (Bayer AG & GE Plastics, USA)
**PMMA (Polymethyl Methacrylate)**	1928 (Hill & Crawford)<br>1933 commercial	1933–36 (Rohm & Haas and ICI 'Perspex')
**PTFE (Polytetrafluoroethylene / Teflon)**	1938 (Roy Plunkett, DuPont USA)	1946 (DuPont)<br>1954 (non-stick pans)
**Biodegradable Polymers (PLA, PHA, PBS, starch blends, etc.)**	1920s-30s (natural)<br>1988 (PHA commercial)<br>1992 (PLA pilot)	1990s (small scale)<br>2000s (commercial)

Table S 2: Global Polymer Production by Decade — Approximate Annual Production (million metric tons)

Polymer	1930	1940	1950	1960	1970	1980	1990	2000	2010	2020
PE	—		~1.0	~8	~25	~42	~58	~80	~110	~120
PP	—	—	~0.01	~2	~8	~15	~25	~43	~62	~79
PS	~0.001	~0.05	~0.5	~2	~6	~10	~14	~18	~20	~14
PET	—	~0.001	~0.1	~1	~4	~12	~22	~38	~55	~82
PVC	~0.005	~0.1	~0.5	~2.5	~10	~15	~22	~30	~40	~46
PU	—	~0.005	~0.1	~0.5	~2	~4	~8	~14	~20	~26
PA	—	~0.01	~0.2	~0.8	~2.5	~3.5	~4.5	~5.5	~6.0	~7.0
PC	—	—	~0.001	~0.1	~0.4	~1.0	~2.0	~3.0	~4.4	~5.5
PMMA	~0.005	~0.05	~0.2	~0.5	~1.0	~1.5	~2.0	~2.8	~3.5	~4.0
PTFE	—	~0.001	~0.005	~0.02	~0.05	~0.08	~0.12	~0.15	~0.20	~0.25
Biodegrada-ble Polymers	—	—	—	—	—	—	~0.01	~0.5	~1.5	~2.2

Note: All values are approximate annual production in million metric tons (Mt/yr) at the representative point within each decade. '—' indicates the polymer was not yet produced commercially at that time. PET figures include polyester fibre; PA figures are primarily fibre production.

Colour coding (Confidence of the data): green = verified/industry data, yellow = estimated from growth rates, orange = rough estimate with high uncertainty.

Table S 3: Key references and data sources

Polymer(s)	Source	Description	Type
All plastics	Geyer, Jambeck & Law (2017)	Production, use, and fate of all plastics ever made. Science Advances 3(7): e1700782. DOI: 10.1126/sciadv.1700782	Peer-reviewed journal article
All plastics	PlasticsEurope / PEMRG	Annual plastics production data 1950–2023. Consultic Marketing & Industrieberatung GmbH. Available at: www.plasticseurope.org	Industry trade association
PE, PP, PVC, PET, PS, PUR	Statista (multiple)	Polymer-specific market volumes and production data. AgileIntel Research (ChemIntel360), GlobalData, Business Wire, Indorama Ventures. statista.com	Aggregated market research
PE	Wikipedia – Polyethylene	Production history: >100 Mt/yr as of 2017. https://en.wikipedia.org/wiki/Polyethylene	Encyclopedia (secondary)
PP	Plastics Insight / INCCA	PP production 2016 ~62 Mt global (Statista/Plastics Insight). INCCA history article: incca.org/articles/history-and-use-polypropylene	Industry / conservation
PVC	Jinhetec / PMC/NCBI	PVC production 2022: ~45.6 Mt. PMC/NCBI peer-reviewed article on PVC risks. DOI: 10.3390/ma17010173 jinhetec.com; pmc.ncbi.nlm.nih.gov	Industry + peer-reviewed
PET	Business Wire / FNF Research / Wikipedia	2014: ~41.6 Mt (Business Wire via Statista). 2020: ~82 Mt incl. fiber (Wikipedia). 2021: >55 Mt (FNF Research). wkaiglobal.com history article.	Multiple industry
PU	Wikipedia – Polyurethane	1960: >45 kt flexible foam. Mid-1980s: 4 Mt. 2019: 25 Mt. Covestro/Statista: 2022 ~25.8 Mt. essentialchemicalindustry.org polyurethane article.	Encyclopedia + industry
PA	Textile Exchange / IVC (German Fiber Assoc.)	1975: 2.49 Mt fiber. 2023: 6.7 Mt fiber. statista.com/statistics/649908. IVC = Industrievereinigung Chemiefaser.	Trade association
PC	Essential Chemical Industry / Statista	2016: ~4.4 Mt global (essentialchemicalindustry.org). 2015 capacity: 4.85 Mt (pc-sheets.com via Statista).	Industry reports
PMMA	Industry reports (various)	Global production ~3.5–4.5 Mt/yr. Limited historic data; estimates from growth rate analysis and market research reports.	Estimated
PTFE	Essential Chemical Industry / Statista / Market Reports World	2013: ~200 kt (Statista). 2015: ~240 kt capacity. 2023: ~240 kt+ (marketreportsworld.com). Discovery history: DuPont 1938.	Industry + market research
Biodegradable	European Bioplastics Association	Annual reports published each December. 2023: 2.18 Mt capacity, ~1.13 Mt biodegradable. 2025: 2.31 Mt capacity. european-bioplastics.org/market	Trade association
Biodegradable	PMC/NCBI – Ahmed et al. 2022	PLA & PHAs review. PLA global production ~190 kt in 2019. PMC article pmc.ncbi.nlm.nih.gov/articles/PMC9033233	Peer-reviewed journal article
Biodegradable	EEA Circularity Metrics Lab	Historical bioplastic capacity data 2010–2023. eea.europa.eu/en/circularity/sectoral-modules/plastics	Government / regulatory
All plastics	Grist / OECD (2022)	Global plastics 2019: 460 Mt. Projections to 2060. grist.org plastics crisis article citing OECD data.	Media citing OECD
